# Identifying regulatory targets of cell cycle transcription factors using gene expression and ChIP-chip data

**DOI:** 10.1186/1471-2105-8-188

**Published:** 2007-06-08

**Authors:** Wei-Sheng Wu, Wen-Hsiung Li, Bor-Sen Chen

**Affiliations:** 1 Lab of Control and Systems Biology, Department of Electrical Engineering, National Tsing Hua University, Hsinchu, 300, Taiwan; 2 Department of Evolution and Ecology, University of Chicago, 1101 East 57^th ^Street, Chicago, IL, 60637, USA; 3 Genomics Research Center, Academia Sinica, Taipei, Taiwan

## Abstract

**Background:**

ChIP-chip data, which indicate binding of transcription factors (TFs) to DNA regions *in vivo*, are widely used to reconstruct transcriptional regulatory networks. However, the binding of a TF to a gene does not necessarily imply regulation. Thus, it is important to develop methods to identify regulatory targets of TFs from ChIP-chip data.

**Results:**

We developed a method, called Temporal Relationship Identification Algorithm (TRIA), which uses gene expression data to identify a TF's regulatory targets among its binding targets inferred from ChIP-chip data. We applied TRIA to yeast cell cycle microarray data and identified many plausible regulatory targets of cell cycle TFs. We validated our predictions by checking the enrichments for functional annotation and known cell cycle genes. Moreover, we showed that TRIA performs better than two published methods (MA-Network and MFA). It is known that co-regulated genes may not be co-expressed. TRIA has the ability to identify subsets of highly co-expressed genes among the regulatory targets of a TF. Different functional roles are found for different subsets, indicating the diverse functions a TF could have. Finally, for a control, we showed that TRIA also performs well for cell-cycle irrelevant TFs.

**Conclusion:**

Finding the regulatory targets of TFs is important for understanding how cells change their transcription program to adapt to environmental stimuli. Our algorithm TRIA is helpful for achieving this purpose.

## Background

By organizing the genes in a genome into transcriptional regulatory modules (TRMs), a living cell can coordinate the activities of many genes and carry out complex functions. Therefore, identifying TRMs is useful for understanding cellular responses to internal and external signals. Advances in high-throughput tools such as DNA microarray [1,2] and chromatin immunoprecipitation-chip (ChIP-chip) [3,4] have made the computational reconstruction of TRMs of a eukaryotic cell possible.

Genome-wide gene expression analysis has been used to investigate TRMs controlling a variety of cellular processes in yeast [5-9]. Clustering and motif-discovering algorithms have been applied to gene expression data to find sets of co-regulated genes and have identified plausible binding motifs of their TFs [7,10,11]. Such approaches have also been expanded to incorporate existing knowledge about the genes, such as cellular functions [12] or promoter sequence motifs [13]. Moreover, some researchers used model-based approaches such as random Boolean networks [14] and Bayesian networks [15,16] to infer regulatory network architectures. However, this approach provides only indirect evidence of genetic regulatory interactions and does not identify the relevant TFs.

On the other hand, the ChIP-chip technique was developed to identify physical interactions between TFs and DNA regions. Using ChIP-chip data, Simon *et al*. [17] investigated how the yeast cell-cycle gene-expression program is regulated by each of nine major transcriptional activators. Lee *et al*. [18] constructed a network of TF-gene interactions and Harbison *et al*. [19] constructed an initial map of yeast's transcriptional regulatory code. However, a weakness in the ChIP-chip technique is that the binding of a TF to a gene does not necessarily imply regulation. A TF may bind to a gene but has no regulatory effect on that gene's expression. Even if a TF does regulate a specific gene, the ChIP-chip data alone does not tell whether the regulation is activation or repression. Hence, additional information is required to solve this ambiguity inherent in ChIP-chip data.

To overcome this problem, several algorithms have been developed to combine gene expression and ChIP-chip data to infer the regulatory targets of a TF. For instance, NCA [20] and MA-Network [21] both use multivariate regression analysis and MFA [22] uses modified factor analysis of gene expression data to classify a TF's binding targets inferred from ChIP-chip data into regulatory and non-regulatory targets. In this paper, we use a different approach to explore the different biological possibilities for the same phenomenon. We develop a method, called Temporal Relationship Identification Algorithm (TRIA), which uses time-lagged correlation analysis between a TF and its binding targets to identify its regulatory targets. Our rationale is that a TF has a high time-lagged correlation with its regulatory targets, but has a low time-lagged correlation with its binding but non-regulatory targets. Time-lagged correlation analysis has the ability to infer causality and directional relationships between genes [23,24]. It has also been used to reconstruct the reaction network of central carbon metabolism [25] and the gene interaction networks of *Synechocystis sp *[26]. Therefore, time-lagged correlation analysis has the potential to be used to identify a TF's regulatory targets from its binding targets which may or may not be regulated by the TF.

## Results

### Identification of the plausible regulatory targets of a TF

Two previous papers [18,19] used a statistical error model to assign a *p*-value to the binding relationship of a TF-gene pair. They found that if *p*-value ≤ 0.001, the binding relationship of a TF-gene pair is of high confidence and can usually be confirmed by gene-specific PCR. Therefore, we include a gene in the set *B*^+ ^if the TF-gene binding *p*-value in the ChIP-chip data is ≤ 0.001, i.e. *B*^+ ^consists of genes that are significantly bound by a TF. Further, a gene in *B*^+ ^is assigned into *B*^+^*R*^+ ^if it has a temporal relationship with the TF but into *B*^+^*R*^- ^otherwise. A TF-gene pair is said to have a temporal relationship if the gene's expression profile is significantly correlated with the TF's regulatory profile possibly with time lags (see Methods). Our hypothesis is that the genes in *B*^+^*R*^+ ^are more likely to be the regulatory targets of a TF than are the genes in *B*^+^*R*^-^. TRIA is developed to classify *B*^+ ^into *B*^+^*R*^+ ^and *B*^+^*R*^-^.

### Only a subset of the binding targets are plausible regulatory targets of a TF

We considered nine cell cycle TFs that have both sizes of *B*^+^*R*^+ ^and *B*^+^*R*^- ^≥ 25 (i.e. at least 25 genes in each group). The number of genes in each group (*B*^+^*R*^+ ^and *B*^+^*R*^-^) is listed in Table 1. On average, 55% of significantly bound genes are identified as the plausible regulatory targets of a TF, similar to the result (58%) of [21], and 64% of the inferred regulatory targets have expression profiles that are positively correlated with the TF's regulatory profile possibly with time lags. Moreover, only 16% of the inferred regulatory targets and the TF are co-expressed (i.e. identified time lag = 0). That is, 84% of the inferred regulatory targets may not be found if we use the conventional correlation analysis that can only check whether a TF-gene pair are co-expressed or not (see Additional file 1 for details). The following analyses were performed to validate our method.

**Table 1:** Classification of the binding targets of a TF into plausible and non-plausible regulatory ones. The numbers of genes in *B*^+^, *B*^+^*R*^+ ^and *B*^+^*R*^- ^are shown for each of the nine cell cycle TFs under study. *B*^+^*R*^+ ^is further divided into two subsets depending on whether the gene's expression profile is positively (*TlC *> 0) or negatively (*TlC *< 0) correlated with the TF's regulatory profile, possibly with time lags (see Additional file 1 for details).

TF	*B*^+^	*B*^+^*R*^+ ^(*TlC *> 0, *TlC *< 0)	*B*^+^*R*^-^
Abf1	247	144 (85,59)	103
Ace2	81	44 (23,21)	37
Cin5	142	69 (35,34)	73
Fkh1	133	96 (62,34)	37
Fkh2	116	90 (60,30)	26
Rap1	147	82 (61,21)	65
Swi4	146	84 (66,18)	62
Swi5	106	42 (32,10)	64
Swi6	144	49 (25,24)	95

### Enrichment for specific functional categories

**Figure 1:**
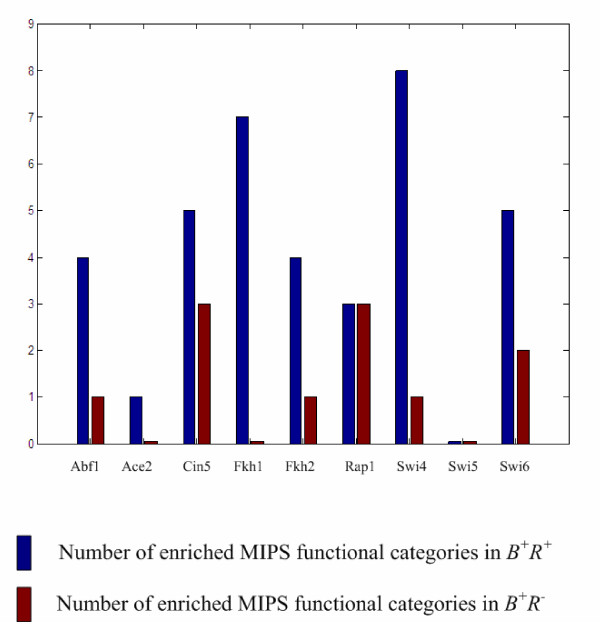
**Enrichment in functional annotation for the cell cycle TFs under study**. The numbers of significantly enriched MIPS functional categories in *B*^+^*R*^+ ^(blue) and *B*^+^*R*^- ^(brown) for each of the nine cell cycle TFs under study are shown.

*B*^+^*R*^+ ^is shown to be more enriched than *B*^+^*R*^- ^for specific MIPS functional categories with adjusted *p*-value < 0.05 (after the Bonferroni correction for multiple tests) using the cumulative hypergeometric distribution (see Additional file 2 for details). In most cases (7/9), except for Rap1 and Swi5, the number of enriched MIPS functional categories in *B*^+^*R*^+ ^is larger than that in *B*^+^*R*^- ^(see Figure 1). This result suggests that our criterion for distinguishing the plausible from non-plausible regulatory targets of a TF is reliable because co-regulated genes should have a greater probability to be involved in the same functional categories than non-co-regulated genes.

### Enrichment for cell cycle genes

We compute the proportions of genes of *B*^+^*R*^+ ^and *B*^+^*R*^- ^that belong to the known cell cycle genes identified by Spellman *et al*. [7]. We then test whether the enrichment of the known cell cycle genes in *B*^+^*R*^+ ^is statistically higher than that in *B*^+^*R*^-^. The cumulative hypergeometric distribution is used to assign a *p*-value for determining the statistical significance (see Appendix for details). In most cases (7/9), except for Abf1 and Ace2, the cell cycle genes are more enriched in *B*^+^*R*^+ ^than in *B*^+^*R*^- ^(see Table 2). This result also suggests that our criterion for distinguishing the plausible from non-plausible regulatory targets of a cell cycle TF is reliable because regulatory targets of a cell cycle TF should be more enriched for the known cell cycle genes than should non-regulatory targets.

**Table 2:** Enrichment of cell cycle genes. The proportions of genes that belong to the 793 cell cycle genes identified by Spellman *et al*. [7] are calculated for *B*^+^*R*^+ ^and *B*^+^*R*^-^. We then test whether the enrichment of the known cell cycle genes in *B*^+^*R*^+ ^is statistically higher than that in *B*^+^*R*^-^. The cumulative hypergeometric distribution is used to determine the statistical significance (see the Appendix for details). In most cases (7/9), except for Abf1 and Ace2, the known cell cycle genes are more enriched in *B*^+^*R*^+ ^than in *B*^+^*R*^-^.

TF	*B*^+^*R*^+^	*B*^+^*R*^-^	*p*-value	(*n*_*a*_, *m*_*a*_, *n*_*b*_, *m*_*b*_)
Abf1	19/144	6/103	0.0439	(144,19,103,6)
Ace2	14/44	7/37	0.1433	(44,14,37,7)
Cin5	24/69	11/73	**0.0055**	(69,24,73,11)
Fkh1	41/96	3/37	**5.9970e-005**	(96,41,37,3)
Fkh2	54/90	0/26	**3.7043e-009**	(90,54,26,0)
Rap1	13/82	2/65	**0.0092**	(82,13,65,2)
Swi4	60/84	15/62	**1.2199e-008**	(84,60,62,15)
Swi5	22/42	14/64	**0.0012**	(42,22,64,14)
Swi6	37/49	42/95	**2.7593e-004**	(49,37,95,42)

Taken together, the results mentioned above convincingly demonstrate that TRIA is a good method for identifying the plausible regulatory targets of a TF from its binding targets.

### Identifying highly co-expressed genes among the plausible regulatory targets of a TF

It is known that co-regulated genes may not be co-expressed [28]. Therefore, it is useful to identify highly co-expressed genes among co-regulated genes because these co-regulated and highly co-expressed genes should be more likely to be simultaneously co-activated or co-repressed by the same TF and involve in the same cellular process.

TRIA has the ability to identify subsets of highly co-expressed genes among the regulatory targets of a TF. First, we use TRIA to identify the plausible regulatory targets (*B*^+^*R*^+^) from the binding targets (*B*^+^) of a TF. Then, we classify *B*^+^*R*^+ ^into subsets *A*_*i *_and *R*_*i*_, where *A*_*i *_(*R*_*i*_) contains all genes whose expression profiles are positively (negatively) correlated with the TF's regulatory profile with a lag of *i *time points. Finally, we test whether the expression coherence of *X*_*i *_is statistically higher than that of *B*^+^*R*^+^, where *X*_*i *_= *A*_*i *_or *R*_*i*_. The expression coherence of genes in a set *G *(i.e. *EC*(*G*)) is defined as the fraction of gene pairs in *G *with a correlation in expression level higher than a threshold *T *[27]. The threshold *T *was determined to be the 95^th ^percentile correlation value of all pairwise correlations between 2000 randomly chosen genes in the yeast genome. Note that 0 ≤ *EC*(*G*) ≤ 1. The cumulative hypergeometric distribution is used to assign a *p*-value for rejecting the null hypothesis *EC*(*X*_*i*_) = *EC*(*B*^+^*R*^+^), where *X*_*i *_= *A*_*i *_or *R*_*i *_(see the Appendix for details).

Table 3 lists all subsets of *X*_*i*_'*s *that contain highly co-expressed genes with *p*-value < 0.001. This result shows that in general several groups of highly co-expressed genes can be extracted from the co-regulated genes, consistent with the result of [28]. These co-regulated and highly co-expressed genes should be more likely to be simultaneously co-activated or co-repressed by the TF and can be used as candidates for further experimental studies. As shown in Table 3, different subsets may have different functional roles, indicating the diverse functions a TF might have.

**Table 3:** Identification of highly co-expressed genes among the regulatory targets of a TF. The expression coherence (*EC*) of *B*^+^*R*^+^, *A*_*i *_and *R*_*i *_are calculated. We then test whether the expression coherence of *X*_*i *_is statistically higher than that of *B*^+^*R*^-^, where *X*_*i *_= *A*_*i *_or *R*_*i*_. The cumulative hypergeometric distribution is used to assign a *p*-value for rejecting the null hypothesis *EC*(*X*_*i*_) = *EC*(*B*^+^*R*^+^). Only those X′is
 MathType@MTEF@5@5@+=feaafiart1ev1aaatCvAUfKttLearuWrP9MDH5MBPbIqV92AaeXatLxBI9gBaebbnrfifHhDYfgasaacH8akY=wiFfYdH8Gipec8Eeeu0xXdbba9frFj0=OqFfea0dXdd9vqai=hGuQ8kuc9pgc9s8qqaq=dirpe0xb9q8qiLsFr0=vr0=vr0dc8meaabaqaciaacaGaaeqabaqabeGadaaakeaacuWGybawgaqbamaaBaaaleaacqWGPbqAaeqaaOGaem4Camhaaa@30F1@ that have *p *< 0.001 (i.e., -log_10 _*p *> 3) are shown (see the Appendix for details). In addition, we show the most enriched MIPS functional category for each *X*_*i*_.

TF(*EC*(*B*^+^*R*^+^))	*X*_*i *_(*EC*(*X*_*i*_); -log_10_(*p*-value))				
Abf1(0.15)	A_1_(0.64;Inf)	A_3_(0.51;Inf)	R_6_(0.34;Inf)		
	PROTEIN WITH BINDING FUNCTION OR COFACTOR REQUIREMENT	TRANSCRIPTION	BIOGENESIS OF CELLULAR COMPONENTS		
Ace2(0.07)	A_3_(0.31;5.11)	A_4_(0.5;3.66)	R_3_(1;3.55)		
	CELL CYCLE AND DNA PROCESSING	REGULATION OF METABOLISM AND PROTEIN FUNCTION	METABOLISM		
Cin5(0.08)	A_0_(0.73;9.14)	A_1_(0.43;6.29)	A_5_(0.61;11.63)	R_0_(0.76;Inf)	R_2_(0.47;4.14)
	ENERGY	CELLULAR TRANSPORT, TRANSPORT FACILITIES AND TRANSPORT ROUTES	METABOLISM	CELLULAR TRANSPORT, TRANSPORT FACILITIES AND TRANSPORT ROUTES	CELLULAR TRANSPORT, TRANSPORT FACILITIES AND TRANSPORT ROUTES
Fkh1(0.12)	A_0_(0.65;11.29)	A_1_(0.49;11.03)	A_2_(0.27;4.18)		
	CELL CYCLE AND DNA PROCESSING	CELL TYPE DIFFERENTIATION	CELL CYCLE AND DNA PROCESSING		
Fkh2(0.16)	A_0_(0.69;Inf)	A_1_(0.7;Inf)	A_2_(0.69;11.44)	A_3_(0.76;8.82)	
	CELL CYCLE AND DNA PROCESSING	CELL CYCLE AND DNA PROCESSING	CELL TYPE DIFFERENTIATION	PROTEIN FATE	
Rap1(0.11)	A_2_(0.58;Inf)	A_4_(0.62;Inf)	A_5_(1;9.46)		
	PROTEIN SYNTHESIS	PROTEIN SYNTHESIS	UNCLASSIFIED PROTEINS		
Swi4(0.2)	A_0_(0.87;Inf)	A_1_(0.6;Inf)	A_2_(0.79;Inf)	A_3_(0.71;6.19)	
	CELL CYCLE AND DNA PROCESSING	METABOLISM	CELL CYCLE AND DNA PROCESSING	CELL CYCLE AND DNA PROCESSING	
Swi5(0.17)	A_0_(1;7.79)	A_2_(0.86;11.36)	A_3_(0.64;7.78)		
	BIOGENESIS OF CELLULAR COMPONENTS	INTERACTION WITH THE ENVIRONMENT	CELL RESCUE, DEFENSE AND VIRULENCE		
Swi6(0.23)	A_0_(0.9;10.18)	A_6_(0.73;4.33)	A_7_(0.75;8.25)	R_2_(0.61;4.76)	
	BIOGENESIS OF CELLULAR COMPONENTS	CELL CYCLE AND DNA PROCESSING	METABOLISM	CELL CYCLE AND DNA PROCESSING	

### Performance comparison with existing methods

To identify the regulatory targets of a TF, Gao *et al*. [21] developed MA-Network that uses multivariate regression analysis of gene expression data and Yu *et al*. [22] developed a modified factor analysis (MFA) approach. We compare the identified regulatory targets of the TFs that are available in our study and at least one of the other two studies. On average, only 53% of our identified regulatory targets are also found by MA-Network and only 31% of our identified regulatory targets are also found by MFA. There is little overlap between the above three studies. This is not surprising biologically since the three methods study different biological possibilities for the same phenomenon. However, since the results of the three methods are not highly congruent, a performance comparison of these three methods should be done. Since a TF has to bind to its regulatory targets to regulate their expressions, enrichment of the high-confidence TF binding motifs among the identified regulatory targets of a TF can be used as a criterion for performance comparison. The high-confidence TF binding motifs were derived using six motif discovery methods, also including the requirement for conservation across at least three of the four related yeast species [19]. Let *S*_1 _(*T*_1_) be the set of regulatory targets of a TF that are identified by TRIA but not by MA-Network (MFA) and *S*_2 _(*T*_2_) be the set of regulatory targets of a TF that are identified by MA-Network (MFA) but not by TRIA. We tested over-representation of the high-confidence TF binding motifs in *S*_1 _and *S*_2 _(*T*_1 _and *T*_2_). The cumulative hypergeometric distribution is used to assign a *p*-value to the motif enrichment (see the Appendix for details). We found that in four of the five (4/5) cases the high-confidence TF binding motifs are enriched in *S*_1 _with *p*-value < 0.001 but only two of the five (2/5) cases in *S*_2 _are enriched (see Table 4). Similarly, we found that in six of the eight (6/8) cases the high-confidence TF binding motifs are enriched in *T*_1 _with *p*-value < 0.001 but none of the eight (0/8) cases in *T*_2 _is enriched (see Table 5). The results show that TRIA has a better ability to identify the regulatory targets of a TF than do MA-Network and MFA.

**Table 4:** Performance comparison of TRIA with MA-Network. We tested over-representation of the high-confidence TF binding motif in *S*_1 _and *S*_2_, where *S*_1 _is the set of regulatory targets of a TF that are identified by TRIA but not by MA-Network and *S*_2 _is the set of regulatory targets of a TF that are identified by MA-Network but not by TRIA. The proportions of genes, whose promoter regions contain the high-confidence TF binding motif is calculated for *S*_1 _and *S*_2_. The cumulative hypergeometric distribution is used to determine the statistical significance of over-representation (see the Appendix for details). In four of the five (4/5) cases the high-confidence TF binding motifs are enriched in *S*_1 _with *p*-value < 0.001 but only two of the five (2/5) cases in *S*_2_.

TF	*S*_1_	*p*-value	*S*_2_	*p*-value
Abf1	46/62	**0**	28/56	**3.0839e-011**
Ace2	2/28	0.0340	2/17	0.0132
Fkh2	17/47	**1.5357e-008**	7/18	**1.8019e-004**
Swi4	16/27	**6.5301e-012**	6/18	0.0021
Swi5	9/25	**2.4141e-004**	7/30	0.0171

**Table 5:** Performance comparison of TRIA with MFA. We tested over-representation of the high-confidence TF binding motif in *T*_1 _and *T*_2_, where *T*_1 _is the set of regulatory targets of a TF that are identified by TRIA but not by MFA and *T*_2 _is the set of regulatory targets of a TF that are identified by MFA but not by TRIA. The proportions of genes, whose promoter regions contain the high-confidence TF binding motif is calculated for *T*_1 _and *T*_2_. The cumulative hypergeometric distribution is used to determine the statistical significance of the over-representation (see the Appendix for details). In six of the eight (6/8) cases the high-confidence TF binding motifs are enriched in *T*_1 _with *p*-value < 0.001 but none of the eight (0/8) cases in *T*_2_.

TF	*T*_1_	*p*-value	*T*_2_	*p*-value
Abf1	75/105	**4.0357e-012**	10/106	0.9042
Ace2	1/31	0.2782	3/35	0.0056
Fkh1	30/64	**3.1252e-007**	5/109	1.0000
Fkh2	20/49	**6.6581e-011**	10/100	0.2038
Rap1	32/72	**1.2579e-011**	7/36	0.0052
Swi4	28/56	**5.3634e-012**	2/36	0.7981
Swi5	7/26	0.0076	4/32	0.3417
Swi6	19/30	**2.4500e-009**	13/72	0.2932

## Discussion

Many researchers used ChIP-chip data to study regulatory networks of the yeast [17-19,29,30]. Most of them (except [29]) regarded that a gene is regulated by a TF if the gene is bound by the TF with a *p*-value ≤ 0.001 in the ChIP-chip data. However, a TF that binds to a gene may have no regulatory effect on that gene. Therefore, additional information is required to solve this uncertainty. TRIA was developed to overcome this problem and was applied to gene expression and ChIP-chip data to identify the plausible regulatory targets of nine cell cycle TFs. The effectiveness of TRIA was validated by statistically testing for the enrichment of functional groups and known cell cycle genes.

Since co-expressed genes are not necessarily co-regulated and vice versa [28], it is important to develop a method that can identify co-regulated genes that are not co-expressed. TRIA has the ability to do this task. Through identifying a TF's binding targets that have temporal relationships with the TF, we can find the TF's regulatory targets that may not be co-expressed. We can further identify subsets of highly co-expressed genes among the inferred regulatory targets according to the identified time lags and regulatory directions. These co-regulated and highly co-expressed genes should be more likely to be simultaneously co-activated or co-repressed by the TF and can be used as candidates for further experimental studies.

TRIA has been successfully used by two previous studies to investigate other biological problems. First, Tsai *et al*. [31] developed TFBSfinder, which utilizes several data sources (DNA sequences, phylogenetic information, microarray data and ChIP-chip data), to identify cell cycle TF binding sites in yeast. TRIA was used to select reliable target genes of a TF in the first step of their algorithm. The target gene selection is a important step that strongly enhances the performance of TFBSfinder [31]. Since the performance of TFBSfinder is shown to be better than three well-known TF binding site identification algorithms (AlignACE, MDscan and MEME) [31], this confirmed that TRIA does have ability to identify the plausible regulatory targets of a TF. Second, Wu *et al*. [32] developed MOFA, which integrates gene expression and ChIP-chip data, to reconstruct transcriptional regulatory modules (TRMs) of the yeast cell cycle. TRIA was used as the first step of MOFA to refine the noisy raw ChIP-chip data and construct a binding score matrix. The quality of the binding score matrix strongly affects the performance of MOFA [32]. The TRMs identified by MOFA was validated by using existing experimental data, enrichment for genes in the same MIPS functional category, known DNA-binding motifs, etc. In addition, MOFA is capable of finding many novel TF-target gene relationships and can determine whether a TF is an activator or/and a repressor [32]. Since MOFA can reconstruct biologically relevant TRMs of the yeast cell cycle, this also attests to the usefulness of TRIA.

**Figure 2:**
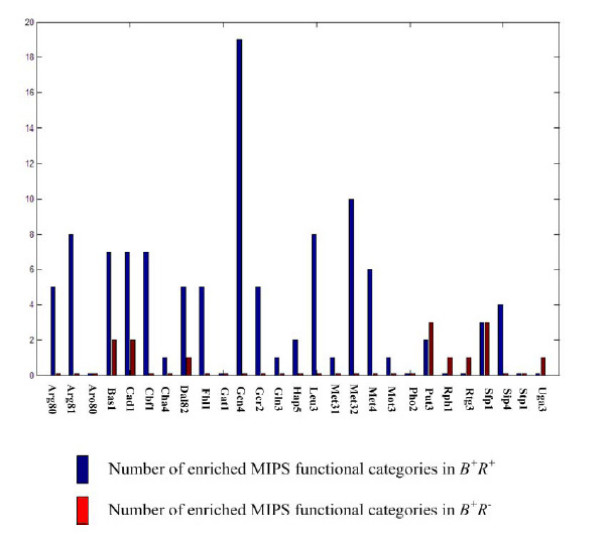
**Enrichment in functional annotation for the stress response TFs under study**. The numbers of significantly enriched MIPS functional categories in *B*^+^*R*^+ ^(blue) and *B*^+^*R*^- ^(brown) for each of the 27 amino acid starved stress TFs under study are shown.

In this paper, TRIA is used to identify regulatory targets of cell cycle TFs. For a control, we show that TRIA can also perform well for cell-cycle irrelevant regulators. In this regard, we apply TRIA to identify regulatory targets of TFs that are activated by amino acid starved stress. The genome-wide gene expression and ChIP-chip data under amino acid starved growth condition are download from [8,19]. As shown in Figure 2, in most of the cases, *B*^+^*R*^+ ^is more enriched than *B*^+^*R*^- ^for specific MIPS functional categories with adjusted *p*-value < 0.05 (after the Bonferroni correction for multiple tests) using the cumulative hypergeometric distribution. This result suggests that TRIA performed well for cell-cycle irrelevant TFs.

The development of TRIA was motivated by two biological observations. First, it is known that TF binding affects gene expression in a nonlinear fashion: below some level it has no effect, and above some level the effect may saturate. This type of behavior can be modeled using a sigmoid function. Therefore, we define a TF's regulatory profile as a sigmoid function of its expression profile as in previous studies [33-35]. Although this may not be true for TFs that are activated at the post-translational stage [20,36], it is not a serious problem for many cell cycle TFs whose expression levels significantly varies with times, indicating that they are under transcriptional control [24,33,34,37-39]. Second, the regulatory effect of a TF on its target genes may not be simultaneous but has a time lag [23,24,26,35,37,38,40-42]. This makes TRIA more general than previous studies [20-22,28] that regard a gene to be regulated by a TF only when the gene's expression profile are co-expressed with the transcription factor activity (TFA) profile. Actually, we found that TRIA performed better than two previous algorithms (MA-Network and MFA) [21,22]. This may result from the fact that TRIA is designed for cell cycle TFs and also considers a time-lagged correlation between a cell cycle TF and its regulatory targets.

In this study, we use time-lagged correlation analysis between a TF and its binding targets to identify its regulatory targets. However, in some cases, TFs may interact with each other and together regulate a group of target genes. This issue will be addressed in the future. We will try to define an overall regulatory profile of a TF complex and apply TRIA to identify target genes that are co-regulated by the same TF complex.

## Conclusion

An algorithm called TRIA is developed to identify the plausible regulatory targets of a TF from its binding targets. Since the binding of a TF to a gene does not necessarily imply regulation, TRIA is used to solve this ambiguity. We validated the effectiveness of TRIA by checking the enrichments for functional annotation and known cell cycle genes. Moreover, the performance of TRIA was shown to be better than two published methods (MA-Network and MFA). Moreover, TRIA has the ability to identify subsets of highly co-expressed genes among the regulatory targets of a TF. In addition, TRIA has been successfully applied to identify high-confidence cell cycle TF binding sites [31] and to reconstruct transcriptional regulatory modules of the yeast cell cycle [32]. Finally, for a control, TRIA is shown to perform well for cell-cycle irrelevant TFs. In conclusion, TRIA can find biologically relevant results and should be useful for systems biology study.

## Methods

### Data sets

Three types of data are used in this study. First, the ChIP-chip data of the cell cycle TFs under the rich media are downloaded from [19]. Second, the gene expression data of the yeast cell cycle are downloaded from [7]. Although it is an old data set, it is still the best cell cycle data set that are available in the public domain. Genes that have only one missing point in their gene expression profiles are reconstructed by the spline algorithm [43], but genes that have more than one missing value in their gene expression profiles or have no ChIP-chip data are excluded. Third, the genome-wide distribution of the high-confidence TF binding motifs was downloaded from [19]. The high-confidence TF binding motifs were derived by using six motif discovery methods, with the requirement for conservation across at least three of four related yeast species [19].

### Temporal Relationship Identification Algorithm (TRIA)

Temporal Relationship Identification Algorithm (TRIA) is developed to identify TF-gene pairs that have a temporal relationship. A cell cycle TF and its binding target are said to have a positively (negatively) temporal relationship if the target gene's expression profile is significantly positively (negatively) correlated with the TF's regulatory profile possibly with a time lag. It is known that TF binding affects gene expression in a nonlinear fashion: below some level it has no effect, and above some level the effect may become saturated. This type of behavior can be modeled using a sigmoid function. Therefore, we define a TF's regulatory profile as a sigmoid function of its expression profile as in previous studies [33-35].

Let x→
 MathType@MTEF@5@5@+=feaafiart1ev1aaatCvAUfKttLearuWrP9MDH5MBPbIqV92AaeXatLxBI9gBaebbnrfifHhDYfgasaacH8akY=wiFfYdH8Gipec8Eeeu0xXdbba9frFj0=OqFfea0dXdd9vqai=hGuQ8kuc9pgc9s8qqaq=dirpe0xb9q8qiLsFr0=vr0=vr0dc8meaabaqaciaacaGaaeqabaqabeGadaaakeaacuWG4baEgaWcaaaa@2E37@ =(*x*_1_, ..., *x*_*N*_) be the gene expression time profile of cell cycle TF *x *and y→
 MathType@MTEF@5@5@+=feaafiart1ev1aaatCvAUfKttLearuWrP9MDH5MBPbIqV92AaeXatLxBI9gBaebbnrfifHhDYfgasaacH8akY=wiFfYdH8Gipec8Eeeu0xXdbba9frFj0=OqFfea0dXdd9vqai=hGuQ8kuc9pgc9s8qqaq=dirpe0xb9q8qiLsFr0=vr0=vr0dc8meaabaqaciaacaGaaeqabaqabeGadaaakeaacuWG5bqEgaWcaaaa@2E39@ = (*y*_1_, ..., *y*_*N*_) be the expression profile of gene *y*. The regulatory profile *RP*(x→
 MathType@MTEF@5@5@+=feaafiart1ev1aaatCvAUfKttLearuWrP9MDH5MBPbIqV92AaeXatLxBI9gBaebbnrfifHhDYfgasaacH8akY=wiFfYdH8Gipec8Eeeu0xXdbba9frFj0=OqFfea0dXdd9vqai=hGuQ8kuc9pgc9s8qqaq=dirpe0xb9q8qiLsFr0=vr0=vr0dc8meaabaqaciaacaGaaeqabaqabeGadaaakeaacuWG4baEgaWcaaaa@2E37@) = (*f*(*x*_1_), ..., *f*(*x*_*N*_)) of TF *x *is defined as a sigmoid function:

$$\begin{array}{cc} f(x_{i})=\frac{1}{1+e^{-(x_{i}-\bar{x})/s)}} & i=1,2,\cdots ,N \end{array}$$

where x¯
 MathType@MTEF@5@5@+=feaafiart1ev1aaatCvAUfKttLearuWrP9MDH5MBPbIqV92AaeXatLxBI9gBaebbnrfifHhDYfgasaacH8akY=wiFfYdH8Gipec8Eeeu0xXdbba9frFj0=OqFfea0dXdd9vqai=hGuQ8kuc9pgc9s8qqaq=dirpe0xb9q8qiLsFr0=vr0=vr0dc8meaabaqaciaacaGaaeqabaqabeGadaaakeaacuWG4baEgaqeaaaa@2E3D@ is the sample mean and *s *is the sample standard deviation of x→
 MathType@MTEF@5@5@+=feaafiart1ev1aaatCvAUfKttLearuWrP9MDH5MBPbIqV92AaeXatLxBI9gBaebbnrfifHhDYfgasaacH8akY=wiFfYdH8Gipec8Eeeu0xXdbba9frFj0=OqFfea0dXdd9vqai=hGuQ8kuc9pgc9s8qqaq=dirpe0xb9q8qiLsFr0=vr0=vr0dc8meaabaqaciaacaGaaeqabaqabeGadaaakeaacuWG4baEgaWcaaaa@2E37@. Compute the correlation between y→
 MathType@MTEF@5@5@+=feaafiart1ev1aaatCvAUfKttLearuWrP9MDH5MBPbIqV92AaeXatLxBI9gBaebbnrfifHhDYfgasaacH8akY=wiFfYdH8Gipec8Eeeu0xXdbba9frFj0=OqFfea0dXdd9vqai=hGuQ8kuc9pgc9s8qqaq=dirpe0xb9q8qiLsFr0=vr0=vr0dc8meaabaqaciaacaGaaeqabaqabeGadaaakeaacuWG5bqEgaWcaaaa@2E39@ and *RP*(x→
 MathType@MTEF@5@5@+=feaafiart1ev1aaatCvAUfKttLearuWrP9MDH5MBPbIqV92AaeXatLxBI9gBaebbnrfifHhDYfgasaacH8akY=wiFfYdH8Gipec8Eeeu0xXdbba9frFj0=OqFfea0dXdd9vqai=hGuQ8kuc9pgc9s8qqaq=dirpe0xb9q8qiLsFr0=vr0=vr0dc8meaabaqaciaacaGaaeqabaqabeGadaaakeaacuWG4baEgaWcaaaa@2E37@) with a lag of *k *time points [24,25]:

r(k)=(∑i=1N−k(yi+k−y¯)(f(xi)−m¯))/(∑i=1N−k(yi+k−y¯)2⋅∑i=1N−k(f(xi)−m¯)2),k=0,1,...,L
 MathType@MTEF@5@5@+=feaafiart1ev1aaatCvAUfKttLearuWrP9MDH5MBPbIqV92AaeXatLxBI9gBaebbnrfifHhDYfgasaacH8akY=wiFfYdH8Gipec8Eeeu0xXdbba9frFj0=OqFfea0dXdd9vqai=hGuQ8kuc9pgc9s8qqaq=dirpe0xb9q8qiLsFr0=vr0=vr0dc8meaabaqaciaacaGaaeqabaqabeGadaaakeaafaqabeqacaaabaWaaSGbaeaacqWGYbGCcqGGOaakcqWGRbWAcqGGPaqkcqGH9aqpdaqadaqaamaaqahabaGaeiikaGIaemyEaK3aaSbaaSqaaiabdMgaPjabgUcaRiabdUgaRbqabaGccqGHsislcuWG5bqEgaqeaiabcMcaPiabcIcaOiabdAgaMjabcIcaOiabdIha4naaBaaaleaacqWGPbqAaeqaaOGaeiykaKIaeyOeI0IafmyBa0MbaebacqGGPaqkaSqaaiabdMgaPjabg2da9iabigdaXaqaaiabd6eaojabgkHiTiabdUgaRbqdcqGHris5aaGccaGLOaGaayzkaaaabaWaaeWaaeaadaGcaaqaamaaqahabaGaeiikaGIaemyEaK3aaSbaaSqaaiabdMgaPjabgUcaRiabdUgaRbqabaGccqGHsislcuWG5bqEgaqeaaWcbaGaemyAaKMaeyypa0JaeGymaedabaGaemOta4KaeyOeI0Iaem4AaSganiabggHiLdGccqGGPaqkdaahaaWcbeqaaiabikdaYaaaaeqaaOGaeyyXIC9aaOaaaeaadaaeWbqaaiabcIcaOiabdAgaMjabcIcaOiabdIha4naaBaaaleaacqWGPbqAaeqaaOGaeiykaKIaeyOeI0IafmyBa0MbaebacqGGPaqkdaahaaWcbeqaaiabikdaYaaaaeaacqWGPbqAcqGH9aqpcqaIXaqmaeaacqWGobGtcqGHsislcqWGRbWAa0GaeyyeIuoaaSqabaaakiaawIcacaGLPaaaaaGaeiilaWcabaGaem4AaSMaeyypa0JaeGimaaJaeiilaWIaeGymaeJaeiilaWIaeiOla4IaeiOla4IaeiOla4IaeiilaWIaemitaWeaaaaa@8825@

where y¯≜(∑i=1N−kyi+k)/(N−k)
 MathType@MTEF@5@5@+=feaafiart1ev1aaatCvAUfKttLearuWrP9MDH5MBPbIqV92AaeXatLxBI9gBaebbnrfifHhDYfgasaacH8akY=wiFfYdH8Gipec8Eeeu0xXdbba9frFj0=OqFfea0dXdd9vqai=hGuQ8kuc9pgc9s8qqaq=dirpe0xb9q8qiLsFr0=vr0=vr0dc8meaabaqaciaacaGaaeqabaqabeGadaaakeaadaWcgaqaaiqbdMha5zaaraGaeSixIa0aaeWaaeaadaaeWbqaaiabdMha5naaBaaaleaacqWGPbqAcqGHRaWkcqWGRbWAaeqaaaqaaiabdMgaPjabg2da9iabigdaXaqaaiabd6eaojabgkHiTiabdUgaRbqdcqGHris5aaGccaGLOaGaayzkaaaabaGaeiikaGIaemOta4KaeyOeI0Iaem4AaSMaeiykaKcaaaaa@4496@, m¯≜(∑i=1N−kf(xi))/(N−k)
 MathType@MTEF@5@5@+=feaafiart1ev1aaatCvAUfKttLearuWrP9MDH5MBPbIqV92AaeXatLxBI9gBaebbnrfifHhDYfgasaacH8akY=wiFfYdH8Gipec8Eeeu0xXdbba9frFj0=OqFfea0dXdd9vqai=hGuQ8kuc9pgc9s8qqaq=dirpe0xb9q8qiLsFr0=vr0=vr0dc8meaabaqaciaacaGaaeqabaqabeGadaaakeaadaWcgaqaaiqbd2gaTzaaraGaeSixIa0aaeWaaeaadaaeWbqaaiabdAgaMjabcIcaOiabdIha4naaBaaaleaacqWGPbqAaeqaaOGaeiykaKcaleaacqWGPbqAcqGH9aqpcqaIXaqmaeaacqWGobGtcqGHsislcqWGRbWAa0GaeyyeIuoaaOGaayjkaiaawMcaaaqaaiabcIcaOiabd6eaojabgkHiTiabdUgaRjabcMcaPaaaaaa@4557@ and *L *is the maximal time lag of the TF's regulatory profile considered. In this study, we set *L *= 8 meaning that we compute the correlation between a gene and a TF with all possible time lags that are less than one cell cycle. The time lag may be interpreted as the time for a TF to have a regulatory effect on a gene.

Then we test the null hypothesis H_0_: *r*(*k*) = 0 and the alternative hypothesis H_1_: *r*(*k*) ≠ 0 by the bootstrap method (see the Appendix) and get a *p*-value *p*(*k*). The time-lagged correlation (*TlC*) of y→
 MathType@MTEF@5@5@+=feaafiart1ev1aaatCvAUfKttLearuWrP9MDH5MBPbIqV92AaeXatLxBI9gBaebbnrfifHhDYfgasaacH8akY=wiFfYdH8Gipec8Eeeu0xXdbba9frFj0=OqFfea0dXdd9vqai=hGuQ8kuc9pgc9s8qqaq=dirpe0xb9q8qiLsFr0=vr0=vr0dc8meaabaqaciaacaGaaeqabaqabeGadaaakeaacuWG5bqEgaWcaaaa@2E39@ and *RP*(x→
 MathType@MTEF@5@5@+=feaafiart1ev1aaatCvAUfKttLearuWrP9MDH5MBPbIqV92AaeXatLxBI9gBaebbnrfifHhDYfgasaacH8akY=wiFfYdH8Gipec8Eeeu0xXdbba9frFj0=OqFfea0dXdd9vqai=hGuQ8kuc9pgc9s8qqaq=dirpe0xb9q8qiLsFr0=vr0=vr0dc8meaabaqaciaacaGaaeqabaqabeGadaaakeaacuWG4baEgaWcaaaa@2E37@) is defined as *r*(*j*) that has the smallest *p*-value (i.e. *TlC*(y→
 MathType@MTEF@5@5@+=feaafiart1ev1aaatCvAUfKttLearuWrP9MDH5MBPbIqV92AaeXatLxBI9gBaebbnrfifHhDYfgasaacH8akY=wiFfYdH8Gipec8Eeeu0xXdbba9frFj0=OqFfea0dXdd9vqai=hGuQ8kuc9pgc9s8qqaq=dirpe0xb9q8qiLsFr0=vr0=vr0dc8meaabaqaciaacaGaaeqabaqabeGadaaakeaacuWG5bqEgaWcaaaa@2E39@, *RP*(x→
 MathType@MTEF@5@5@+=feaafiart1ev1aaatCvAUfKttLearuWrP9MDH5MBPbIqV92AaeXatLxBI9gBaebbnrfifHhDYfgasaacH8akY=wiFfYdH8Gipec8Eeeu0xXdbba9frFj0=OqFfea0dXdd9vqai=hGuQ8kuc9pgc9s8qqaq=dirpe0xb9q8qiLsFr0=vr0=vr0dc8meaabaqaciaacaGaaeqabaqabeGadaaakeaacuWG4baEgaWcaaaa@2E37@)) = *r*(*j*) if *p*(*j*) ≤ *p*(*k*) ∀*k *≠ *j*). Note that -1 ≤ *TlC*(y→
 MathType@MTEF@5@5@+=feaafiart1ev1aaatCvAUfKttLearuWrP9MDH5MBPbIqV92AaeXatLxBI9gBaebbnrfifHhDYfgasaacH8akY=wiFfYdH8Gipec8Eeeu0xXdbba9frFj0=OqFfea0dXdd9vqai=hGuQ8kuc9pgc9s8qqaq=dirpe0xb9q8qiLsFr0=vr0=vr0dc8meaabaqaciaacaGaaeqabaqabeGadaaakeaacuWG5bqEgaWcaaaa@2E39@, *RP*(x→
 MathType@MTEF@5@5@+=feaafiart1ev1aaatCvAUfKttLearuWrP9MDH5MBPbIqV92AaeXatLxBI9gBaebbnrfifHhDYfgasaacH8akY=wiFfYdH8Gipec8Eeeu0xXdbba9frFj0=OqFfea0dXdd9vqai=hGuQ8kuc9pgc9s8qqaq=dirpe0xb9q8qiLsFr0=vr0=vr0dc8meaabaqaciaacaGaaeqabaqabeGadaaakeaacuWG4baEgaWcaaaa@2E37@)) ≤ 1. Two possible temporal relationships between y→
 MathType@MTEF@5@5@+=feaafiart1ev1aaatCvAUfKttLearuWrP9MDH5MBPbIqV92AaeXatLxBI9gBaebbnrfifHhDYfgasaacH8akY=wiFfYdH8Gipec8Eeeu0xXdbba9frFj0=OqFfea0dXdd9vqai=hGuQ8kuc9pgc9s8qqaq=dirpe0xb9q8qiLsFr0=vr0=vr0dc8meaabaqaciaacaGaaeqabaqabeGadaaakeaacuWG5bqEgaWcaaaa@2E39@ and *RP*(x→
 MathType@MTEF@5@5@+=feaafiart1ev1aaatCvAUfKttLearuWrP9MDH5MBPbIqV92AaeXatLxBI9gBaebbnrfifHhDYfgasaacH8akY=wiFfYdH8Gipec8Eeeu0xXdbba9frFj0=OqFfea0dXdd9vqai=hGuQ8kuc9pgc9s8qqaq=dirpe0xb9q8qiLsFr0=vr0=vr0dc8meaabaqaciaacaGaaeqabaqabeGadaaakeaacuWG4baEgaWcaaaa@2E37@) can be identified by TRIA: y→
 MathType@MTEF@5@5@+=feaafiart1ev1aaatCvAUfKttLearuWrP9MDH5MBPbIqV92AaeXatLxBI9gBaebbnrfifHhDYfgasaacH8akY=wiFfYdH8Gipec8Eeeu0xXdbba9frFj0=OqFfea0dXdd9vqai=hGuQ8kuc9pgc9s8qqaq=dirpe0xb9q8qiLsFr0=vr0=vr0dc8meaabaqaciaacaGaaeqabaqabeGadaaakeaacuWG5bqEgaWcaaaa@2E39@ and *RP*(x→
 MathType@MTEF@5@5@+=feaafiart1ev1aaatCvAUfKttLearuWrP9MDH5MBPbIqV92AaeXatLxBI9gBaebbnrfifHhDYfgasaacH8akY=wiFfYdH8Gipec8Eeeu0xXdbba9frFj0=OqFfea0dXdd9vqai=hGuQ8kuc9pgc9s8qqaq=dirpe0xb9q8qiLsFr0=vr0=vr0dc8meaabaqaciaacaGaaeqabaqabeGadaaakeaacuWG4baEgaWcaaaa@2E37@) are (1) positively correlated with a lag of *j *time points if *TlC*(y→
 MathType@MTEF@5@5@+=feaafiart1ev1aaatCvAUfKttLearuWrP9MDH5MBPbIqV92AaeXatLxBI9gBaebbnrfifHhDYfgasaacH8akY=wiFfYdH8Gipec8Eeeu0xXdbba9frFj0=OqFfea0dXdd9vqai=hGuQ8kuc9pgc9s8qqaq=dirpe0xb9q8qiLsFr0=vr0=vr0dc8meaabaqaciaacaGaaeqabaqabeGadaaakeaacuWG5bqEgaWcaaaa@2E39@, *RP*(x→
 MathType@MTEF@5@5@+=feaafiart1ev1aaatCvAUfKttLearuWrP9MDH5MBPbIqV92AaeXatLxBI9gBaebbnrfifHhDYfgasaacH8akY=wiFfYdH8Gipec8Eeeu0xXdbba9frFj0=OqFfea0dXdd9vqai=hGuQ8kuc9pgc9s8qqaq=dirpe0xb9q8qiLsFr0=vr0=vr0dc8meaabaqaciaacaGaaeqabaqabeGadaaakeaacuWG4baEgaWcaaaa@2E37@)) = *r*(*j*) > 0 &*p*(*j*) ≤ *p*_*Threshold *_and (2) negatively correlated with a lag of *j *time points if *TlC*(y→
 MathType@MTEF@5@5@+=feaafiart1ev1aaatCvAUfKttLearuWrP9MDH5MBPbIqV92AaeXatLxBI9gBaebbnrfifHhDYfgasaacH8akY=wiFfYdH8Gipec8Eeeu0xXdbba9frFj0=OqFfea0dXdd9vqai=hGuQ8kuc9pgc9s8qqaq=dirpe0xb9q8qiLsFr0=vr0=vr0dc8meaabaqaciaacaGaaeqabaqabeGadaaakeaacuWG5bqEgaWcaaaa@2E39@, *RP*(x→
 MathType@MTEF@5@5@+=feaafiart1ev1aaatCvAUfKttLearuWrP9MDH5MBPbIqV92AaeXatLxBI9gBaebbnrfifHhDYfgasaacH8akY=wiFfYdH8Gipec8Eeeu0xXdbba9frFj0=OqFfea0dXdd9vqai=hGuQ8kuc9pgc9s8qqaq=dirpe0xb9q8qiLsFr0=vr0=vr0dc8meaabaqaciaacaGaaeqabaqabeGadaaakeaacuWG4baEgaWcaaaa@2E37@)) = *r*(*j*) < 0 &*p*(*j*) ≤ *p*_*Threshold*_. The *p*_*Threshold *_is chosen to ensure that we have at most a 5% false discovery rate (FDR) [44]. We may consider that TF *x*, after a lag of *j *time points, activates (represses) gene *y *if y→
 MathType@MTEF@5@5@+=feaafiart1ev1aaatCvAUfKttLearuWrP9MDH5MBPbIqV92AaeXatLxBI9gBaebbnrfifHhDYfgasaacH8akY=wiFfYdH8Gipec8Eeeu0xXdbba9frFj0=OqFfea0dXdd9vqai=hGuQ8kuc9pgc9s8qqaq=dirpe0xb9q8qiLsFr0=vr0=vr0dc8meaabaqaciaacaGaaeqabaqabeGadaaakeaacuWG5bqEgaWcaaaa@2E39@ and *RP*(x→
 MathType@MTEF@5@5@+=feaafiart1ev1aaatCvAUfKttLearuWrP9MDH5MBPbIqV92AaeXatLxBI9gBaebbnrfifHhDYfgasaacH8akY=wiFfYdH8Gipec8Eeeu0xXdbba9frFj0=OqFfea0dXdd9vqai=hGuQ8kuc9pgc9s8qqaq=dirpe0xb9q8qiLsFr0=vr0=vr0dc8meaabaqaciaacaGaaeqabaqabeGadaaakeaacuWG4baEgaWcaaaa@2E37@) are positively (negatively) correlated with a lag of *j *time points.

## Appendix

### Statistical test used in Table 2

We want to test whether the enrichment of the known cell cycle genes (identified in [7]) in *B*^+^*R*^+ ^is statistically higher than that in *B*^+^*R*^-^. Following Banerjee and Zhang [27], a model based on hypergeometric distribution is used.

We calculate:

P(ma,mb,na,nb)=(nama)(nbmb)(na+nbma+mb)=(nama)(N−naM−ma)(NM)
 MathType@MTEF@5@5@+=feaafiart1ev1aaatCvAUfKttLearuWrP9MDH5MBPbIqV92AaeXatLxBI9gBaebbnrfifHhDYfgasaacH8akY=wiFfYdH8Gipec8Eeeu0xXdbba9frFj0=OqFfea0dXdd9vqai=hGuQ8kuc9pgc9s8qqaq=dirpe0xb9q8qiLsFr0=vr0=vr0dc8meaabaqaciaacaGaaeqabaqabeGadaaakeaacqWGqbaucqGGOaakcqWGTbqBdaWgaaWcbaGaemyyaegabeaakiabcYcaSiabd2gaTnaaBaaaleaacqWGIbGyaeqaaOGaeiilaWIaemOBa42aaSbaaSqaaiabdggaHbqabaGccqGGSaalcqWGUbGBdaWgaaWcbaGaemOyaigabeaakiabcMcaPiabg2da9maalaaabaWaaeWaaeaafaqabeGabaaabaGaemOBa42aaSbaaSqaaiabdggaHbqabaaakeaacqWGTbqBdaWgaaWcbaGaemyyaegabeaaaaaakiaawIcacaGLPaaadaqadaqaauaabeqaceaaaeaacqWGUbGBdaWgaaWcbaGaemOyaigabeaaaOqaaiabd2gaTnaaBaaaleaacqWGIbGyaeqaaaaaaOGaayjkaiaawMcaaaqaamaabmaabaqbaeqabiqaaaqaaiabd6gaUnaaBaaaleaacqWGHbqyaeqaaOGaey4kaSIaemOBa42aaSbaaSqaaiabdkgaIbqabaaakeaacqWGTbqBdaWgaaWcbaGaemyyaegabeaakiabgUcaRiabd2gaTnaaBaaaleaacqWGIbGyaeqaaaaaaOGaayjkaiaawMcaaaaacqGH9aqpdaWcaaqaamaabmaabaqbaeqabiqaaaqaaiabd6gaUnaaBaaaleaacqWGHbqyaeqaaaGcbaGaemyBa02aaSbaaSqaaiabdggaHbqabaaaaaGccaGLOaGaayzkaaWaaeWaaeaafaqabeGabaaabaGaemOta4KaeyOeI0IaemOBa42aaSbaaSqaaiabdggaHbqabaaakeaacqWGnbqtcqGHsislcqWGTbqBdaWgaaWcbaGaemyyaegabeaaaaaakiaawIcacaGLPaaaaeaadaqadaqaauaabeqaceaaaeaacqWGobGtaeaacqWGnbqtaaaacaGLOaGaayzkaaaaaaaa@7461@

where *N *= *n*_*a *_+ *n*_*b*_, *M *= *m*_*a *_+ *m*_*b*_, *n*_*a*_(*n*_*b*_) is the number of genes in *B*^+^*R*^+ ^(*B*^+^*R*^-^), *m*_*a*_(*m*_*b*_) is the number of the known cell cycle genes in *B*^+^*R*^+ ^(*B*^+^*R*^-^), and

(nama)≜na!ma!(na−ma)!
 MathType@MTEF@5@5@+=feaafiart1ev1aaatCvAUfKttLearuWrP9MDH5MBPbIqV92AaeXatLxBI9gBaebbnrfifHhDYfgasaacH8akY=wiFfYdH8Gipec8Eeeu0xXdbba9frFj0=OqFfea0dXdd9vqai=hGuQ8kuc9pgc9s8qqaq=dirpe0xb9q8qiLsFr0=vr0=vr0dc8meaabaqaciaacaGaaeqabaqabeGadaaakeaadaqadaqaauaabeqaceaaaeaacqWGUbGBdaWgaaWcbaGaemyyaegabeaaaOqaaiabd2gaTnaaBaaaleaacqWGHbqyaeqaaaaaaOGaayjkaiaawMcaaiablYLianaalaaabaGaemOBa42aaSbaaSqaaiabdggaHbqabaGccqGGHaqiaeaacqWGTbqBdaWgaaWcbaGaemyyaegabeaakiabcgcaHiabcIcaOiabd6gaUnaaBaaaleaacqWGHbqyaeqaaOGaeyOeI0IaemyBa02aaSbaaSqaaiabdggaHbqabaGccqGGPaqkcqGGHaqiaaaaaa@45FC@

Then, we consider all possible combinations of *x*_*a*_, *x*_*b *_such that ∑i={a,b}xi=∑i={a,b}mi=M
 MathType@MTEF@5@5@+=feaafiart1ev1aaatCvAUfKttLearuWrP9MDH5MBPbIqV92AaeXatLxBI9gBaebbnrfifHhDYfgasaacH8akY=wiFfYdH8Gipec8Eeeu0xXdbba9frFj0=OqFfea0dXdd9vqai=hGuQ8kuc9pgc9s8qqaq=dirpe0xb9q8qiLsFr0=vr0=vr0dc8meaabaqaciaacaGaaeqabaqabeGadaaakeaadaaeqbqaaiabdIha4naaBaaaleaacqWGPbqAaeqaaaqaaiabdMgaPjabg2da9iabcUha7jabdggaHjabcYcaSiabdkgaIjabc2ha9bqab0GaeyyeIuoakiabg2da9maaqafabaGaemyBa02aaSbaaSqaaiabdMgaPbqabaGccqGH9aqpcqWGnbqtaSqaaiabdMgaPjabg2da9iabcUha7jabdggaHjabcYcaSiabdkgaIjabc2ha9bqab0GaeyyeIuoaaaa@4BC6@ and sum all probabilities calculated as above where *x*_*a *_≥ *m*_*a*_, which is taken as the *p*-value for rejecting the null hypothesis that enrichment of the known cell cycle genes in *B*^+^*R*^+ ^is not statistically higher than that in *B*^+^*R*^-^.

p=P(xa≥ma)=∑xa≥ma(naxa)(N−naM−xa)(NM)=1−∑xa=0ma−1(naxa)(N−naM−xa)(NM)
 MathType@MTEF@5@5@+=feaafiart1ev1aaatCvAUfKttLearuWrP9MDH5MBPbIqV92AaeXatLxBI9gBaebbnrfifHhDYfgasaacH8akY=wiFfYdH8Gipec8Eeeu0xXdbba9frFj0=OqFfea0dXdd9vqai=hGuQ8kuc9pgc9s8qqaq=dirpe0xb9q8qiLsFr0=vr0=vr0dc8meaabaqaciaacaGaaeqabaqabeGadaaakeaacqWGWbaCcqGH9aqpcqWGqbaucqGGOaakcqWG4baEdaWgaaWcbaGaemyyaegabeaakiabgwMiZkabd2gaTnaaBaaaleaacqWGHbqyaeqaaOGaeiykaKIaeyypa0ZaaabuaeaadaWcaaqaamaabmaabaqbaeqabiqaaaqaaiabd6gaUnaaBaaaleaacqWGHbqyaeqaaaGcbaGaemiEaG3aaSbaaSqaaiabdggaHbqabaaaaaGccaGLOaGaayzkaaWaaeWaaeaafaqabeGabaaabaGaemOta4KaeyOeI0IaemOBa42aaSbaaSqaaiabdggaHbqabaaakeaacqWGnbqtcqGHsislcqWG4baEdaWgaaWcbaGaemyyaegabeaaaaaakiaawIcacaGLPaaaaeaadaqadaqaauaabeqaceaaaeaacqWGobGtaeaacqWGnbqtaaaacaGLOaGaayzkaaaaaaWcbaGaemiEaG3aaSbaaWqaaiabdggaHbqabaWccqGHLjYScqWGTbqBdaWgaaadbaGaemyyaegabeaaaSqab0GaeyyeIuoakiabg2da9iabigdaXiabgkHiTmaaqahabaWaaSaaaeaadaqadaqaauaabeqaceaaaeaacqWGUbGBdaWgaaWcbaGaemyyaegabeaaaOqaaiabdIha4naaBaaaleaacqWGHbqyaeqaaaaaaOGaayjkaiaawMcaamaabmaabaqbaeqabiqaaaqaaiabd6eaojabgkHiTiabd6gaUnaaBaaaleaacqWGHbqyaeqaaaGcbaGaemyta0KaeyOeI0IaemiEaG3aaSbaaSqaaiabdggaHbqabaaaaaGccaGLOaGaayzkaaaabaWaaeWaaeaafaqabeGabaaabaGaemOta4eabaGaemyta0eaaaGaayjkaiaawMcaaaaaaSqaaiabdIha4naaBaaameaacqWGHbqyaeqaaSGaeyypa0JaeGimaadabaGaemyBa02aaSbaaWqaaiabdggaHbqabaWccqGHsislcqaIXaqma0GaeyyeIuoaaaa@8145@

### Statistical test used in Table 3

The expression coherence (*EC*) of sets *B*^+^*R*^+^, *A*_*i *_and *R*_*i *_are calculated, where *A*_*i *_(*R*_*i*_) contains all genes in *B*^+^*R*^+ ^whose expression profiles are positively (negatively) correlated with the TF's regulatory profile with a lag of *i *time points.

We want to test whether the expression coherence of *X*_*i *_is statistically higher than that of *B*^+^*R*^-^, where *X*_*i *_= *A*_*i *_or *R*_*i*_. The *p*-value for rejecting the null hypothesis *EC*(*X*_*i*_) = *EC*(*B*^+^*R*^+^) (the alternative hypothesis is *EC*(*X*_*i*_) > *EC*(*B*^+^*R*^+^)) is defined as in Equation (*A*1). *N *is the number of gene pairs in *B*^+^*R*^+^, *M *is the number of gene pairs in *X*_*i*_, where *X*_*i *_= *A*_*i *_or *R*_*i*_, *n*_*a *_is the number of gene pairs in *B*^+^*R*^+ ^that have correlations higher than the threshold *T*, and *m*_*a *_is the number of gene pairs in *X*_*i *_that have correlations higher than the threshold *T*.

### Statistical tests used in Tables 4 and 5

The proportions of genes whose promoter regions contain the high-confidence TF binding motif are calculated for *S*_1 _(*T*_1_) and *S*_2 _(*T*_2_), where *S*_1 _(*T*_1_) is the set of regulatory targets of a TF that are identified by TRIA but not by MA-Network (MFA) and *S*_2_(*T*_2_) is the set of regulatory targets of a TF that are identified by MA-Network (MFA) but not by TRIA. Only 5 TFs (Abf1, Ace2, Fkh2, Swi4, Swi5) are studied for both TRIA and MA-Network. Only 8 TFs (Abf1, Ace2, Fkh1, Fkh2, Rap1, Swi4, Swi5, Swi6) are studied for both TRIA and MFA.

The high-confidence TF binding motifs were derived by using six motif discovery methods, under the requirement for conservation across at least three of the four related yeast species [19]. The yeast genome has 6229 ORFs. Only 817 genes contain Abf1 binding sites, 65 genes contains Ace2 binding sites, 461 genes contain Fkh2 binding sites, 501 genes contain Swi4 binding sites, 575 genes contain Swi5 binding sites, 1181 genes contain Fkh1 binding sites, 379 genes contain Rap1 binding sites, and 946 genes contain Swi6 binding sites [19].

We tested over-representation of the high-confidence TF binding motif in *S*_1 _(*T*_1_) and *S*_2 _(*T*_2_). The cumulative hypergeometric distribution is used to determine the statistical significance. The *p*-value is defined as in Equation (*A*1), where *N *= 6229 is the number of genes in the yeast genome, *M *is the number of genes in *G*, where *G *= *S*_1 _(*T*_1_) or *S*_2_(*T*_2_) (e.g. *M *= 62 for Abf1 if *G *= *S*_1 _and *M *= 56 for Abf1 if *G *= *S*_2_; *M *= 72 for Rap1 if *G *= *T*_1 _and *M *= 36 for Rap1 if *G *= *T*_2_), *n*_*a *_is the number of genes in the yeast genome that contain binding sites of a TF under study (e.g. *n*_*a *_= 817 for Abf1; *n*_*a *_= 379 for Rap1) and *m*_*a *_is the number of genes in *G *that contain binding sites of a TF under study (e.g. *m*_*a *_= 46 for Abf1 if *G *= *S*_1 _and *m*_*a *_= 28 for Abf1 if *G *= *S*_2_; *m*_*a *_= 32 for Rap1 if *G *= *T*_1 _and *m*_*a *_= 7 for Rap1 if *G *= *T*_2_).

### The bootstrap method for testing the statistical significance of the difference between *r*(*k*) and 0

We observed *N*-*k *pairs of observations, *Z *= {**z**_i_: i = 1, ..., *N*-*k *and **z**_i _= (*f*(*x*_*i*_), *y*_*i*+*k*_)}. The correlation coefficient from the sample is calculated and denoted as

r(k)=(∑i=1N−k(yi+k−y¯)(f(xi)−m¯))/(∑i=1N−k(yi+k−y¯)2⋅∑i=1N−k(f(xi)−m¯)2),k=0,1,2,⋯
 MathType@MTEF@5@5@+=feaafiart1ev1aaatCvAUfKttLearuWrP9MDH5MBPbIqV92AaeXatLxBI9gBaebbnrfifHhDYfgasaacH8akY=wiFfYdH8Gipec8Eeeu0xXdbba9frFj0=OqFfea0dXdd9vqai=hGuQ8kuc9pgc9s8qqaq=dirpe0xb9q8qiLsFr0=vr0=vr0dc8meaabaqaciaacaGaaeqabaqabeGadaaakeaafaqabeqacaaabaWaaSGbaeaacqWGYbGCcqGGOaakcqWGRbWAcqGGPaqkcqGH9aqpdaqadaqaamaaqahabaWaaeWaaeaacqWG5bqEdaWgaaWcbaGaemyAaKMaey4kaSIaem4AaSgabeaakiabgkHiTiqbdMha5zaaraaacaGLOaGaayzkaaWaaeWaaeaacqWGMbGzcqGGOaakcqWG4baEdaWgaaWcbaGaemyAaKgabeaakiabcMcaPiabgkHiTiqbd2gaTzaaraaacaGLOaGaayzkaaaaleaacqWGPbqAcqGH9aqpcqaIXaqmaeaacqWGobGtcqGHsislcqWGRbWAa0GaeyyeIuoaaOGaayjkaiaawMcaaaqaamaabmaabaWaaOaaaeaadaaeWbqaamaabmaabaGaemyEaK3aaSbaaSqaaiabdMgaPjabgUcaRiabdUgaRbqabaGccqGHsislcuWG5bqEgaqeaaGaayjkaiaawMcaamaaCaaaleqabaGaeGOmaidaaaqaaiabdMgaPjabg2da9iabigdaXaqaaiabd6eaojabgkHiTiabdUgaRbqdcqGHris5aaWcbeaakiabgwSixpaakaaabaWaaabCaeaadaqadaqaaiabdAgaMjabcIcaOiabdIha4naaBaaaleaacqWGPbqAaeqaaOGaeiykaKIaeyOeI0IafmyBa0MbaebaaiaawIcacaGLPaaadaahaaWcbeqaaiabikdaYaaaaeaacqWGPbqAcqGH9aqpcqaIXaqmaeaacqWGobGtcqGHsislcqWGRbWAa0GaeyyeIuoaaSqabaaakiaawIcacaGLPaaaaaGaeiilaWcabaGaem4AaSMaeyypa0JaeGimaaJaeiilaWIaeGymaeJaeiilaWIaeGOmaiJaeiilaWIaeS47IWeaaaaa@868A@

where y¯≜(∑i=1N−kyi+k)/(N−k)
 MathType@MTEF@5@5@+=feaafiart1ev1aaatCvAUfKttLearuWrP9MDH5MBPbIqV92AaeXatLxBI9gBaebbnrfifHhDYfgasaacH8akY=wiFfYdH8Gipec8Eeeu0xXdbba9frFj0=OqFfea0dXdd9vqai=hGuQ8kuc9pgc9s8qqaq=dirpe0xb9q8qiLsFr0=vr0=vr0dc8meaabaqaciaacaGaaeqabaqabeGadaaakeaadaWcgaqaaiqbdMha5zaaraGaeSixIa0aaeWaaeaadaaeWbqaaiabdMha5naaBaaaleaacqWGPbqAcqGHRaWkcqWGRbWAaeqaaaqaaiabdMgaPjabg2da9iabigdaXaqaaiabd6eaojabgkHiTiabdUgaRbqdcqGHris5aaGccaGLOaGaayzkaaaabaWaaeWaaeaacqWGobGtcqGHsislcqWGRbWAaiaawIcacaGLPaaaaaaaaa@446D@, m¯≜(∑i=1N−kf(xi))/(N−k)
 MathType@MTEF@5@5@+=feaafiart1ev1aaatCvAUfKttLearuWrP9MDH5MBPbIqV92AaeXatLxBI9gBaebbnrfifHhDYfgasaacH8akY=wiFfYdH8Gipec8Eeeu0xXdbba9frFj0=OqFfea0dXdd9vqai=hGuQ8kuc9pgc9s8qqaq=dirpe0xb9q8qiLsFr0=vr0=vr0dc8meaabaqaciaacaGaaeqabaqabeGadaaakeaadaWcgaqaaiqbd2gaTzaaraGaeSixIa0aaeWaaeaadaaeWbqaaiabdAgaMjabcIcaOiabdIha4naaBaaaleaacqWGPbqAaeqaaOGaeiykaKcaleaacqWGPbqAcqGH9aqpcqaIXaqmaeaacqWGobGtcqGHsislcqWGRbWAa0GaeyyeIuoaaOGaayjkaiaawMcaaaqaamaabmaabaGaemOta4KaeyOeI0Iaem4AaSgacaGLOaGaayzkaaaaaaaa@452E@ and -1 ≤ *r*(*k*) ≤ 1. It is aimed to use these observations to test if *r*(*k*) is different from 0 significantly. Suppose the null hypothesis is H_0_: *r*(*k*)= 0 and the alternative hypothesis is H_1_: *r*(*k*) ≠ 0. We will apply the bootstrap method to perform this hypothesis testing based on the observations. Keeping the pair relationship of these *N*-*k *pairs to maintain the dependence between (*f*(*x*_*i*_), *y*_*i*+*k*_), **z**_i _are sampled with replacement *N*-*k *times to form a bootstrap sample, Z* = {**z**_i_*: i = 1, ..., *N*-*k *and **z**_i_* belongs to *Z*}. The correlation coefficient from the bootstrap sample Z* is computed and denoted as r*(*k*), -1 ≤ r*(*k*) ≤ 1. Repeat the resampling procedure B times, we will observed *r*_1_*(*k*), *r*_2_*(*k*), ..., *r*_B_*(*k*). These bootstrap correlation coefficients are sorted to be −1≤r(1)∗(k)≤r(2)∗(k)≤...≤r(B)∗(k)≤1
 MathType@MTEF@5@5@+=feaafiart1ev1aaatCvAUfKttLearuWrP9MDH5MBPbIqV92AaeXatLxBI9gBaebbnrfifHhDYfgasaacH8akY=wiFfYdH8Gipec8Eeeu0xXdbba9frFj0=OqFfea0dXdd9vqai=hGuQ8kuc9pgc9s8qqaq=dirpe0xb9q8qiLsFr0=vr0=vr0dc8meaabaqaciaacaGaaeqabaqabeGadaaakeaacqGHsislcqaIXaqmcqGHKjYOcqWGYbGCdaqhaaWcbaGaeiikaGIaeGymaeJaeiykaKcabaGaey4fIOcaaOGaeiikaGIaem4AaSMaeiykaKIaeyizImQaemOCai3aa0baaSqaaiabcIcaOiabikdaYiabcMcaPaqaaiabgEHiQaaakiabcIcaOiabdUgaRjabcMcaPiabgsMiJkabc6caUiabc6caUiabc6caUiabgsMiJkabdkhaYnaaDaaaleaacqGGOaakcqWGcbGqcqGGPaqkaeaacqGHxiIkaaGccqGGOaakcqWGRbWAcqGGPaqkcqGHKjYOcqaIXaqmaaa@539F@. Then, the (1-α) two-sided percentile interval is given by [r(B×α/2)∗(k), r(B×(1−α/2))∗(k)
 MathType@MTEF@5@5@+=feaafiart1ev1aaatCvAUfKttLearuWrP9MDH5MBPbIqV92AaeXatLxBI9gBaebbnrfifHhDYfgasaacH8akY=wiFfYdH8Gipec8Eeeu0xXdbba9frFj0=OqFfea0dXdd9vqai=hGuQ8kuc9pgc9s8qqaq=dirpe0xb9q8qiLsFr0=vr0=vr0dc8meaabaqaciaacaGaaeqabaqabeGadaaakeaacqWGYbGCdaqhaaWcbaGaeiikaGIaemOqaiKaey41aqlcciGae8xSdeMaei4la8IaeGOmaiJaeiykaKcabaGaey4fIOcaaOGaeiikaGIaem4AaSMaeiykaKIaeiilaWIaeeiiaaIaemOCai3aa0baaSqaaiabcIcaOiabdkeacjabgEna0kabcIcaOiabigdaXiabgkHiTiab=f7aHjabc+caViabikdaYiabcMcaPiabcMcaPaqaaiabgEHiQaaakiabcIcaOiabdUgaRjabcMcaPaaa@4DC6@] in this case [45]. If this percentile interval does not contain 0, then the null hypothesis is rejected at the significance level of α. Otherwise, the data fail to reject the null hypothesis at the significance level of α. Since the *p*-value is the smallest value of α for which the null hypothesis will be rejected based on the observation, the *p*-value for this test is estimated by the following:

p⌢(k)=2×min⁡{p⌢+(k),1−p⌢+(k)},where p⌢+(k)=∑i=1BI{ri∗(k)≥0}/B,
 MathType@MTEF@5@5@+=feaafiart1ev1aaatCvAUfKttLearuWrP9MDH5MBPbIqV92AaeXatLxBI9gBaebbnrfifHhDYfgasaacH8akY=wiFfYdH8Gipec8Eeeu0xXdbba9frFj0=OqFfea0dXdd9vqai=hGuQ8kuc9pgc9s8qqaq=dirpe0xb9q8qiLsFr0=vr0=vr0dc8meaabaqaciaacaGaaeqabaqabeGadaaakeaafaqabeqacaaabaGafmiCaaNbambacqGGOaakcqWGRbWAcqGGPaqkcqGH9aqpcqaIYaGmcqGHxdaTcyGGTbqBcqGGPbqAcqGGUbGBcqGG7bWEcuWGWbaCgaWeamaaBaaaleaacqGHRaWkaeqaaOGaeiikaGIaem4AaSMaeiykaKIaeiilaWIaeGymaeJaeyOeI0IafmiCaaNbambadaWgaaWcbaGaey4kaScabeaakiabcIcaOiabdUgaRjabcMcaPiabc2ha9jabcYcaSaqaaiabbEha3jabbIgaOjabbwgaLjabbkhaYjabbwgaLjabbccaGiqbdchaWzaataWaaSbaaSqaaiabgUcaRaqabaGccqGGOaakcqWGRbWAcqGGPaqkcqGH9aqpdaaeWbqaaiabdMeajjabcUha7jabdkhaYnaaDaaaleaacqWGPbqAaeaacqGHxiIkaaGccqGGOaakcqWGRbWAcqGGPaqkcqGHLjYScqaIWaamcqGG9bqFcqGGVaWlcqWGcbGqcqGGSaalaSqaaiabdMgaPjabg2da9iabigdaXaqaaiabdkeacbqdcqGHris5aaaaaaa@7106@

where **I**{·} is the indicator function whose value is one when the event is true and zero otherwise.

## Authors' contributions

WSW developed the algorithm, performed the simulation and wrote the manuscript. WHL and BSC gave the research topic, provided essential guidance and revised the manuscript. All authors read and approved the final manuscript.

## Supplementary Material

Additional file 1 Supplementary Table 1 Click here for file

Additional file 2 Supplementary Table 2 Click here for file
