# Supplementary material for: Identifying regulatory targets of cell cycle transcription factors using gene expression and ChIP-chip data
Source: BMC Bioinformatics. 2007 Jun 8;8:188. doi: 10.1186/1471-2105-8-188 (PMC1906835; doi:10.1186/1471-2105-8-188)
Supplement: Additional file 2 — Supplementary Table 2 [file 1471-2105-8-188-S2.pdf]

Supplementary Table 2 Enriched MIPS functional categories found in regulatory targets and non-regulatory targets of a TF

TF+ (no.): regulatory targets of the TF
TF- (no.): non-regulatory targets of the TF
no.: number of enriched MIPS functional categories found
p_{cutoff}: the p-value chosen to make the adjusted p-value < 0.05 (after Bonferroni correction for multiple hypotheses tests)

Abf1+ (4) (p_{cutoff} = 3.4965e-004)

FUNCTIONAL CATEGORY	YOUR GENE MATCHES	GENOME MATCHES	P-VALUE
16.03.03 RNA binding	15 entries (10.4%) (YAL043c YCL011c YDL208w YDR195w YDR312w YGL222c YGR128c YJR093c YKR081c YLR222c YMR061w YMR093w YNL118c YOL077c YPR129w) *annotated-FunCats*	189 entries (2.81%) *annotated-FunCats*	1.10e-05
16.03 nucleic acid binding	20 entries (13.8%) (YAL043c YCL011c YDL208w YDR195w YDR312w YDR404c YGL222c YGR128c YJR063w YJR093c YKR081c YLR222c YMR061w YMR093w YNL118c YNL312w YOL077c YOR116c YPR129w YPR187w) *annotated-FunCats*	340 entries (5.05%) *annotated-FunCats*	3.27e-05
16 PROTEIN WITH BINDING	41 entries (28.4%) (YAL043c YBL079w	1041 entries (15.4%)	4.42e-05

**FUNCTION OR COFACTOR REQUIREMENT (structural or catalytic)**	YBR109c YCL011c YDL116w YDL208w YDR130c YDR195w YDR312w YDR404c YGL222c YGR119c YGR128c YGR270w YHR077c YIL138c YJL008c YJL111w YJR063w YJR093c YKL007w YKR081c YLL034c YLR222c YLR229c YMR061w YMR092c YMR093w YMR129w YNL037c YNL118c YNL312w YNR038w YOL077c YOR056c YOR057w YOR116c YOR117w YPL242c YPR129w YPR187w) ***annotated-FunCats***	***annotated-FunCats***	
**11.04 RNA processing**	21 entries (14.5%) (YAL043c YCL031c YDL208w YDR195w YDR280w YDR463w YGL222c YGR128c YGR129w YJR093c YKL078w YKL172w YKR081c YLR222c YMR061w YMR093w YNL118c YNR038w YOR310c YPL012w YPR104c) ***annotated-FunCats***	434 entries (6.45%) ***annotated-FunCats***	3.35e-04

Abf1- (1) (p_{cutoff} = 4.0650e-004)

FUNCTIONAL CATEGORY	YOUR GENE MATCHES	GENOME MATCHES	P-VALUE
14 PROTEIN FATE (folding, modification, destination)	37 entries (35.9%) (YBL007c YBR110w YBR283c YDL122w YDL159w YDL190c YDR283c YDR295c YDR329c YDR330w YDR422c YGL229c YGR231c YGR232w YHR161c YHR200w YIL031w YJL062w YJR062c YKL016c YKL135c YKL190w YKL195w YKL196c YKR082w YLR024c YLR025w YLR096w YLR396c YNL121c YNL183c YNL243w YOL068c YOL076w YPL037c YPR176c YPR178w) *annotated-FunCats*	1142 entries (16.9%) *annotated-FunCats*	2.49e-06

Ace2+ (1) (p_{cutoff} = 7.0423e-004)

FUNCTIONAL CATEGORY	YOUR GENE MATCHES	GENOME MATCHES	P-VALUE
32.01 stress response	10 entries (22.7%) (YDR011w YER125w YJL159w YJR147w YKR042w YML007w YML100w YMR261c YNL098c YNL241c) *annotated-FunCats*	449 entries (6.67%) *annotated-FunCats*	5.03e-04

Ace2- (0) (p_{cutoff} = 0.0010)

FUNCTIONAL CATEGORY	YOUR GENE MATCHES	GENOME MATCHES	P-VALUE

Cin5+ (5) (p_{cutoff} = 5.8824e-004)

FUNCTIONAL CATEGORY	YOUR GENE MATCHES	GENOME MATCHES	P-VALUE
20.01 transported compounds (substrates)	21 entries (30.4%) (YBL030c YDR343c YDR345c YDR508c YIL170w YIL171w YIR001c YJL219w YJR132w YJR158w YKR066c YLL061w YLR034c YLR081w YML116w YMR246w YMR319c YOR049c YOR306c YOR317w YPR124w) *annotated-FunCats*	588 entries (8.74%) *annotated-FunCats*	2.05e-07|
20.01.03 C-compound and carbohydrate transport	8 entries (11.5%) (YDR343c YDR345c YIL170w YIL171w YJL219w YJR158w YLR081w YOR306c) *annotated-FunCats*	71 entries (1.05%) *annotated-FunCats*	5.17e-07|
20.01.03.01 sugar transport	5 entries (7.24%) (YDR343c YDR345c YJL219w YJR158w YLR081w) *annotated-FunCats*	31 entries (0.46%) *annotated-FunCats*	1.36e-05|
20 CELLULAR TRANSPORT, TRANSPORT FACILITATION AND TRANSPORT ROUTES	24 entries (34.7%) (YBL030c YDR343c YDR345c YDR508c YIL118w YIL170w YIL171w YIR001c YJL219w YJR132w YJR158w YKR066c YKR067w YLL052c	1035 entries (15.3%) *annotated-FunCats*	5.19e-05|

	YLL061w YLR034c YLR081w YML116w YMR246w YMR319c YOR049c YOR306c YOR317w YPR124w) *annotated-FunCats*		
20.09.18 cellular import	8 entries (11.5%) (YDR343c YDR345c YIL170w YJL219w YJR158w YLR081w YMR319c YPR124w) *annotated-FunCats*	151 entries (2.24%) *annotated-FunCats*	1.41e-04|

Cin5- (3) (p_{cutoff} = 6.8493e-004)

FUNCTIONAL CATEGORY	YOUR GENE MATCHES	GENOME MATCHES	P-VALUE
01 METABOLISM	31 entries (42.4%) (YDL037c YDL168w YDR300c YEL046c YEL052w YEL053c YER073w YGR144w YGR180c YHR047c YHR128w YJL031c YJL221c YJR102c YJR103w YKL110c YKL132c YKL182w YLL062c YLR044c YLR228c YLR304c YML131w YMR283c YOL116w YOL151w YOL157c YOR047c YPR035w YPR065w YPR184w) *annotated-FunCats*	1506 entries (22.3%) *annotated-FunCats*	9.51e-05|
01.01.03 assimilation of ammonia,	5 entries (6.84%) (YDR300c YEL053c YKL182w YLR304c	47 entries (0.69%) *annotated-FunCats*	1.41e-04|

metabolism of the glutamate group	YPR035w) *annotated-FunCats*		
01.05 C-compound and carbohydrate metabolism	15 entries (20.5%) (YDL037c YDL168w YER073w YHR047c YJL221c YJR102c YKL110c YLR044c YLR304c YMR283c YOL116w YOL157c YOR047c YPR035w YPR184w) *annotated-FunCats*	505 entries (7.51%) *annotated-FunCats*	2.74e-04|

Fkh1+ (7) (p_{cutoff} = 4.7170e-004)

FUNCTIONAL CATEGORY	YOUR GENE MATCHES	GENOME MATCHES	P-VALUE
10.03 cell cycle	25 entries (26.0%) (YBR078w YBR109c YDL017w YDR108w YDR325w YDR451c YER122c YFL037w YGL028c YGL116w YGR092w YGR098c YHR119w YHR143w YJR091c YKR054c YLR131c YMR076c YMR078c YMR198w YPL139c YPL140c YPL155c YPR075c YPR119w) *annotated-FunCats*	652 entries (9.69%) *annotated-FunCats*	2.85e-06|
10.03.01 mitotic cell cycle and cell cycle control	18 entries (18.7%) (YBR109c YDL017w YER122c YFL037w YGL116w YGR092w YGR098c YHR119w YJR091c YKR054c YLR131c YMR076c YMR078c YMR198w YPL140c YPL155c YPR075c YPR119w) *annotated-FunCats*	446 entries (6.63%) *annotated-FunCats*	4.77e-05|

01.05.01.03.02 polysaccharide biosynthesis	6 entries (6.25%) (YBR110w YLR300w YMR215w YOL030w YPL227c YPR035w) *annotated-FunCats*	51 entries (0.75%) *annotated-FunCats*	7.76e-05|
10.03.01.01.11 mitosis	6 entries (6.25%) (YBR109c YFL037w YGL116w YKR054c YMR198w YPL155c) *annotated-FunCats*	51 entries (0.75%) *annotated-FunCats*	7.76e-05|
10.03.01.01 mitotic cell cycle	10 entries (10.4%) (YBR109c YFL037w YGL116w YKR054c YLR131c YMR076c YMR078c YMR198w YPL155c YPR119w) *annotated-FunCats*	165 entries (2.45%) *annotated-FunCats*	1.10e-04|
10.03.04 nuclear and chromosomal cycle	8 entries (8.33%) (YBR109c YDR325w YFL037w YGR092w YKR054c YMR076c YMR078c YPL155c) *annotated-FunCats*	111 entries (1.65%) *annotated-FunCats*	1.70e-04|
10 CELL CYCLE AND DNA PROCESSING	28 entries (29.1%) (YBR078w YBR109c YDL017w YDR108w YDR325w YDR451c YER070w YER122c YER125w YFL037w YGL028c YGL116w YGR092w YGR098c YHR119w YHR143w YJR091c YKR054c YLR131c YMR076c YMR078c YMR198w YPL116w YPL139c YPL140c YPL155c YPR075c YPR119w) *annotated-FunCats*	1006 entries (14.9%) *annotated-FunCats*	2.55e-04|

Fkh1- (0) (p_{cutoff} = 8.6207e-004)

FUNCTIONAL CATEGORY	YOUR GENE MATCHES	GENOME MATCHES	P-VALUE

Fkh2+ (4) (p_{cutoff} = 4.8077e-004)

FUNCTIONAL CATEGORY	YOUR GENE MATCHES	GENOME MATCHES	P-VALUE
10 CELL CYCLE AND DNA PROCESSING	36 entries (40%) (YAR018c YBL032w YBR009c YBR010w YBR038w YBR078w YBR133c YBR135w YDL017w YDR146c YDR150w YDR451c YER070w YER095w YER125w YGL116w YGL183c YGR092w YHL024w YHR031c YHR143w YHR152w YIL123w YJR091c YKR042w YLR131c YLR286c YLR399c YML064c YMR076c YMR198w YMR199w YNL172w YPL116w YPL155c YPR119w) *annotated-FunCats*	1006 entries (14.9%) *annotated-FunCats*	5.43e-09
10.03 cell cycle	27 entries (30%) (YAR018c YBR038w YBR078w YBR133c YBR135w YDL017w YDR146c YDR150w YDR451c YER095w YGL116w YGR092w YHL024w YHR143w YHR152w YIL123w YJR091c YKR042w YLR131c YLR286c YML064c YMR076c YMR198w YMR199w YNL172w YPL155c YPR119w) *annotated-FunCats*	652 entries (9.69%) *annotated-FunCats*	4.81e-08
10.03.01 mitotic	19 entries (21.1%)	446 entries (6.63%)	4.91e-06

cell cycle and cell cycle control	(YBR133c YBR135w YDL017w YDR146c YDR150w YGL116w YGR092w YHR152w YIL123w YJR091c YKR042w YLR131c YML064c YMR076c YMR198w YMR199w YNL172w YPL155c YPR119w) *annotated-FunCats*	*annotated-FunCats*	
10.03.01.01 mitotic cell cycle	11 entries (12.2%) (YBR133c YDR146c YGL116w YHR152w YLR131c YML064c YMR076c YMR198w YNL172w YPL155c YPR119w) *annotated-FunCats*	165 entries (2.45%) *annotated-FunCats*	1.08e-05|

Fkh2- (1) (p_{cutoff} = 0.0014)

FUNCTIONAL CATEGORY	YOUR GENE MATCHES	GENOME MATCHES	P-VALUE
16.17.09 heavy metal binding (Cu, Fe, Zn)	2 entries (7.69%) (YBR037c YGL256w) *annotated-FunCats*	8 entries (0.11%) *annotated-FunCats*	3.97e-04|

Rap1+ (3) (p_{cutoff} = 8.1967e-004)

FUNCTIONAL CATEGORY	YOUR GENE MATCHES	GENOME MATCHES	P-VALUE
12.01.01 ribosomal proteins	32 entries (39.0%) (YBR084c-a YBR181c YBR189w YBR191w YDL075w YDL082w YDL136w YDR025w YDR064w YDR312w YDR450w YDR471w YDR500c YEL054c YER074w YER117w YGL031c	245 entries (3.64%) *annotated-FunCats*	1.49e-25|

	YGL103w YGL123w YGR034w YGR118w YHR203c YIL018w YKL180w YLR048w YLR388w YLR448w YML063w YOL127w YOR096w YOR234c YPL143w) ***annotated-FunCats***		
**12.01 ribosome biogenesis**	**33 entries (40.2%) (YBR084c-a YBR181c YBR189w YBR191w YDL075w YDL082w YDL136w YDR025w YDR064w YDR312w YDR450w YDR471w YDR500c YEL054c YER074w YER117w YGL031c YGL103w YGL123w YGR034w YGR118w YHR203c YIL018w YKL180w YLR048w YLR388w YLR448w YML063w YNL163c YOL127w YOR096w YOR234c YPL143w)** ***annotated-FunCats***	**308 entries (4.58%)** ***annotated-FunCats***	**1.44e-23**
**12 PROTEIN SYNTHESIS**	**35 entries (42.6%) (YBR084c-a YBR181c YBR189w YBR191w YDL075w YDL082w YDL136w YDR025w YDR064w YDR312w YDR450w YDR471w YDR500c YEL054c YER074w YER117w YFL022c YGL031c YGL103w YGL123w YGR034w YGR118w YHR203c YIL018w YKL180w YLR048w YLR388w YLR448w YML063w YNL163c YOL127w YOR096w YOR234c YPL143w YPR080w)** ***annotated-FunCats***	**479 entries (7.12%)** ***annotated-FunCats***	**1.64e-19**

Rap1- (3) (p_{cutoff} = 7.5758e-004)

FUNCTIONAL CATEGORY	YOUR GENE MATCHES	GENOME MATCHES	P-VALUE
12.01.01 ribosomal proteins	30 entries (46.1%) (YDL184c YDL191w YDR418w YGL030w YGL189c YGR148c YHR021c YHR141c YIL133c YIL148w YJL136c YJL177w YJL189w YJL190c YJL191w YKL006w YLR333c YLR344w YMR242c YNL069c YNL096c YNL162w YNL302c YOL039w YOL040c YOR293w YOR312c YPL131w YPR102c YPR132w) *annotated-FunCats*	245 entries (3.64%) *annotated-FunCats*	1.17e-26|
12.01 ribosome biogenesis	30 entries (46.1%) (YDL184c YDL191w YDR418w YGL030w YGL189c YGR148c YHR021c YHR141c YIL133c YIL148w YJL136c YJL177w YJL189w YJL190c YJL191w YKL006w YLR333c YLR344w YMR242c YNL069c YNL096c YNL162w YNL302c YOL039w YOL040c YOR293w YOR312c YPL131w YPR102c YPR132w) *annotated-FunCats*	308 entries (4.58%) *annotated-FunCats*	1.19e-23|
12 PROTEIN SYNTHESIS	33 entries (50.7%) (YDL184c YDL188c YDL191w YDR418w YGL030w YGL189c YGR148c YHR021c YHR141c YIL133c YIL148w YJL136c YJL177w YJL189w YJL190c YJL191w YKL006w YLL039c YLR333c YLR344w YMR242c YNL069c YNL096c YNL162w YNL302c YOL039w YOL040c	479 entries (7.12%) *annotated-FunCats*	2.04e-21|

	YOR293w YOR302w YOR312c YPL131w YPR102c YPR132w) *annotated-FunCats*		

Swi4+ (8) (p_{cutoff} = 5.3763e-004)

FUNCTIONAL CATEGORY	YOUR GENE MATCHES	GENOME MATCHES	P-VALUE
40 CELL FATE	13 entries (15.4%) (YDR309c YGR014w YGR152c YGR221c YHR061c YHR149c YIL123w YJL187c YKL008c YLR300w YLR332w YNL298w YOL113w) *annotated-FunCats*	272 entries (4.04%) *annotated-FunCats*	2.77e-05|
42.01 cell wall	11 entries (13.0%) (YBR078w YDL055c YEL040w YJL158c YJL186w YKL096w YKL096w-a YLR300w YLR332w YMR306w YMR307w) *annotated-FunCats*	215 entries (3.19%) *annotated-FunCats*	6.60e-05|
42 BIOGENESIS OF CELLULAR COMPONENTS	24 entries (28.5%) (YBR078w YDL055c YDR309c YDR507c YEL040w YGR014w YGR152c YGR221c YHR061c YHR149c YIL123w YJL158c YJL186w YJL187c YJL194w YKL096w YKL096w-a YLR300w YLR332w YLR439w YMR306w YMR307w YNL298w YOR373w)	860 entries (12.7%) *annotated-FunCats*	8.68e-05|

	annotated-FunCats		
**10 CELL CYCLE AND DNA PROCESSING**	26 entries (30.9%) (YBL002w YBL003c YBR078w YCR065w YDL127w YDR224c YDR225w YDR451c YDR507c YER070w YER111c YGR109c YIL123w YJL187c YJL194w YLR332w YML027w YMR199w YNL298w YOL012c YOL113w YOR372c YOR373w YPL126w YPL127c YPR119w) *annotated-FunCats*	1006 entries (14.9%) *annotated-FunCats*	1.46e-04|
**40.01 cell growth / morphogenesis**	11 entries (13.0%) (YDR309c YGR014w YGR152c YGR221c YHR061c YHR149c YJL187c YLR300w YLR332w YNL298w YOL113w) *annotated-FunCats*	238 entries (3.53%) *annotated-FunCats*	1.64e-04|
**10.03 cell cycle**	19 entries (22.6%) (YBR078w YCR065w YDL127w YDR451c YDR507c YER111c YGR109c YIL123w YJL187c YJL194w YLR332w YML027w YMR199w YNL298w YOL113w YOR372c YOR373w YPL126w YPR119w) *annotated-FunCats*	652 entries (9.69%) *annotated-FunCats*	3.33e-04|
**10.03.01 mitotic cell cycle and cell cycle control**	15 entries (17.8%) (YDL127w YDR507c YER111c YGR109c YIL123w	446 entries (6.63%) *annotated-FunCats*	3.54e-04|

	YJL187c YJL194w YLR332w YML027w YMR199w YOL113w YOR372c YOR373w YPL126w YPR119w) *annotated-FunCats*		
43.01.03.05 budding, cell polarity and filament formation	12 entries (14.2%) (YBR161w YDR309c YDR507c YGR014w YGR152c YGR221c YHR061c YHR149c YJL158c YJL187c YNL298w YPR119w) *annotated-FunCats*	314 entries (4.66%) *annotated-FunCats*	4.76e-04|

Swi4- (1) (p_{cutoff} = 5.9524e-004)

FUNCTIONAL CATEGORY	YOUR GENE MATCHES	GENOME MATCHES	P-VALUE
40.10 cell death	3 entries (4.83%) (YDL126c YDR227w YKR042w) *annotated-FunCats*	18 entries (0.26%) *annotated-FunCats*	5.52e-04|

Swi5+ (0) (p_{cutoff} = 8.6207e-004)

FUNCTIONAL CATEGORY	YOUR GENE MATCHES	GENOME MATCHES	P-VALUE

Swi5- (0) (p_{cutoff} = 6.8493e-004)

FUNCTIONAL CATEGORY	YOUR GENE MATCHES	GENOME MATCHES	P-VALUE

Swi6+ (5) (p_{cutoff} = 6.7568e-004)

FUNCTIONAL CATEGORY	YOUR GENE MATCHES	GENOME MATCHES	P-VALUE
10 CELL CYCLE AND DNA PROCESSING	22 entries (44.8%) (YCR065w YDL003w YDR113c YDR224c YDR225w YDR451c YDR507c YER070w YER111c YGR109c YIL026c YIL123w YJL194w YKL113c YLR103c YLR332w YNL273w YNL298w YOR074c YOR372c YOR373w YPR075c) *annotated-FunCats*	1006 entries (14.9%) *annotated-FunCats*	4.91e-07|
10.03 cell cycle	17 entries (34.6%) (YCR065w YDL003w YDR113c YDR451c YDR507c YER111c YGR109c YIL026c YIL123w YJL194w YLR103c YLR332w YNL273w YNL298w YOR372c YOR373w YPR075c) *annotated-FunCats*	652 entries (9.69%) *annotated-FunCats*	1.60e-06|
10.03.01 mitotic cell cycle and cell cycle control	14 entries (28.5%) (YDL003w YDR113c YDR507c YER111c YGR109c YIL026c YIL123w YJL194w YLR103c YLR332w YNL273w YOR372c YOR373w YPR075c) *annotated-FunCats*	446 entries (6.63%) *annotated-FunCats*	2.04e-06|
40 CELL FATE	9 entries (18.3%) (YDR309c YGR221c YHR061c YHR149c YIL123w YJL115w YKL113c YLR332w	272 entries (4.04%) *annotated-FunCats*	1.26e-04|

	YNL298w) *annotated-FunCats*		
**10.01.03 DNA synthesis and replication**	7 entries (14.2%) (YER070w YGR109c YJL194w YKL113c YLR103c YNL273w YOR074c) *annotated-FunCats*	179 entries (2.66%) *annotated-FunCats*	2.81e-04|

Swi6- (2) (p_{cutoff} = 5.0505e-004)

FUNCTIONAL CATEGORY	YOUR GENE MATCHES	GENOME MATCHES	P-VALUE
**42.01 cell wall**	12 entries (12.6%) (YBR078w YDL055c YJL158c YJL184w YJL186w YKL096w-a YLR110c YLR300w YLR342w YMR165c YMR307w YPR159w) *annotated-FunCats*	215 entries (3.19%) *annotated-FunCats*	4.42e-05|
**10.03.01 mitotic cell cycle and cell cycle control**	16 entries (16.8%) (YDL017w YDL101c YDL127w YHR152w YJL187c YKR042w YML027w YMR076c YMR199w YMR262w YNL289w YOL113w YPL024w YPL126w YPR119w YPR120c) *annotated-FunCats*	446 entries (6.63%) *annotated-FunCats*	4.52e-04|
